# Transmural Remodeling of Cardiac Microstructure in Aged Spontaneously Hypertensive Rats by Diffusion Tensor MRI

**DOI:** 10.3389/fphys.2020.00265

**Published:** 2020-03-31

**Authors:** Archontis Giannakidis, Grant T. Gullberg

**Affiliations:** ^1^School of Science and Technology, Nottingham Trent University, Nottingham, United Kingdom; ^2^Life Sciences Division, Lawrence Berkeley National Laboratory, Berkeley, CA, United States; ^3^National Heart & Lung Institute, Imperial College London, London, United Kingdom; ^4^Department of Radiology and Biomedical Imaging, University of California, San Francisco, San Francisco, CA, United States

**Keywords:** myocardium, microstructure, remodeling, left ventricle, diffusion tensor MRI, hypertension

## Abstract

The long-standing high blood pressure (also known as hypertension) overworks the heart. Microstructural remodeling is a key factor of hypertensive heart disease progression. Diffusion tensor magnetic resonance imaging (DT-MRI) is a powerful tool for the rapid noninvasive nondestructive delineation of the cardiomyocyte organization. The spontaneously hypertensive rat (SHR) is a well-established model of genetic hypertension. The goal of this study was to employ high-resolution DT-MRI and the SHR animal model to assess the transmural layer-specific remodeling of myocardial microstructure associated with hypertension. *Ex vivo* experiments were performed on excised formalin-fixed hearts of aged SHRs (*n* = 4) and age-matched controls (*n* = 4). The DT-MRI-derived fractional anisotropy (FA), longitudinal diffusivity (λ_*L*_), transversal diffusivity (λ_*T*_), and mean diffusivity (MD) served as the readout parameters investigated at three transmural zones (i.e., endocardium, mesocardium, and epicardium). The helix angles (HAs) of the aggregated cardiomyocytes and the orientation of laminar sheetlets were also studied. Compared with controls, the SHRs exhibited decreased epicardial FA, while FA changes in the other two transmural regions were insignificant. No substantial differences were observed in the diffusivity parameters and the transmural course of HAs between the two groups. A consistent distribution pattern of laminar sheetlet orientation was not identified for either group. Our findings are in line with the known cellular microstructure from early painstaking histological studies. Biophysical explanations of the study outcomes are provided. In conclusion, our experimental findings indicate that the epicardial microstructure is more vulnerable to high blood pressure leading to more pronounced changes in this region during remodeling. DT-MRI is well-suited for elucidating these alterations. The revealed transmural nonuniformity of myocardial reorganization may shed light on the mechanisms of the microstructure-function relationship in hypertension progression. Our results provide insights into the management of patients with systemic arterial hypertension, thus prevent the progression toward heart failure. The findings of this study should be acknowledged by electromechanical models of the heart that simulate the specific cardiac pathology.

## 1. Introduction

Elevated blood pressure in the arteries (also known as hypertension) is a serious condition that can lead to heart failure, stroke, kidney failure, and blindness among others^1^. More than 1 in 5 adults live with hypertension all around the globe, and complications from persistent high blood pressure account for 12.8% of the total of all deaths worldwide every year^2^. On top of that, the related disability puts a huge burden on medical care budgets. The treatment of hypertension is an important healthcare priority throughout the world^3^.

Focussing on the heart, the chronic elevated arterial pressure adversely affects the cardiac function by compromising the organ's ability to work as a pump which escalates into heart failure. But a key determinant of the heart's pumping function is its unique three-dimensional (3D) microstructural organization (Arts et al., 1979). Therefore, and from a clinical perspective, depicting the remodeling of cardiomyocyte arrangement in hypertension is of vital significance, as this would provide us with a novel insight on the structure-function relationship allowing us to better interpret and model the cardiac behavior in this pathology.

The development of experimental pre-clinical models of left ventricular pressure overload has been very helpful for the study of the microstructural remodeling in hypertension (Lerman et al., 2005). The spontaneously hypertensive rat (SHR) (Okamoto and Aoki, 1963), which exhibits a type of hypertension that is analogous to essential hypertension in humans, is by far the most popular animal model. Early descriptions of the cardiac tissue alterations in the SHR model were based on gross dissection and microscopy (Kawamura et al., 1976; Engelmann et al., 1987). However, and in spite of being very enlightening with regard to cardiomyocyte alignment, this conventional approach is burdensome, tedious, destructive, inherently two-dimensional (2D), susceptible to processing artifacts, and of low spatial resolution (Hsu et al., 2010). Clearly, there is great clinical utility in acquiring the myocardial microstructural remodeling in hypertension by finding a way around the limitations of histology.

Diffusion tensor magnetic resonance imaging (DT-MRI) has emerged (Basser et al., 1994) as a powerful non-invasive non-destructive modality that infers the orientation-dependent microstructure of tissues such as the myocardium by relating the preferential self-diffusion of water molecules to the MRI signal attenuation. Compared to histology, DT-MRI provides far more measurement points in much shorter acquisition times (Winslow et al., 2000). To date, DT-MRI has been employed to assess myocardial microstructure in humans (Reese et al., 1995) and animal models (Healy et al., 2011; Teh et al., 2017), as well as to characterize cardiac pathologies like ischemia (Wu et al., 2006). There are several studies (Hales et al., 2011, 2012; Gilbert et al., 2012; Bernus et al., 2015; Teh et al., 2016, 2017) which have applied DT-MRI to rat hearts.

Given that some stimuli in hypertension have been shown (McCrossan et al., 2004) to produce a transmurally-varied cellular response, in this study we use aged SHRs and DT-MRI to test the hypothesis that the impact of long-standing elevated blood pressure on the cardiomyocyte arrangement depends upon the transmural depth. Because of the inherently low signal-to-noise ratio (SNR) and motion artifacts that currently hamper the high-spatial-resolution DT-MRI of the beating heart within a realistic time frame (Hsu et al., 2010), our investigation was performed *ex vivo*.

The findings of this study are expected to deeepen our understanding of the mechanisms by which microstructural changes make for the progressive deterioration in cardiac function in hypertensive disease. This understanding is currently weak (Wang et al., 2016). The results are also anticipated to enhance our ability to design therapeutic strategies for the effective management of patients with systemic arterial hypertension, and thus prevent the progression toward heart failure. Finally, our findings will also be useful in the development of more accurate subject-specific image-driven electromechanical models of the hypertensive heart. As a matter of fact, few recent modeling studies (Wang et al., 2015, 2016) of the rodent ventricle physiology have already recognized the lack of high-resolution cardiac microstructure data that could be used as input toward constructing constitutive modeling frameworks of the passive myocardium in hypertension.

## 2. Materials and Methods

### 2.1. Study Population and Animal Model

The study population comprised 4 male SHRs, and 4 sex-matched Wistar Kyoto rats (WKYs) that served as the controls. SHR (Okamoto and Aoki, 1963) is a well-established genetic model of hypertension. It clones many crucial attributes of the progression of human hypertensive heart disease toward decompensated heart failure, whereas its treatment using angiotensin-converting-enzyme inhibitors is as effective as in humans (Cingolani et al., 2003). Male SHRs were chosen because they have significantly higher blood pressure than the female ones (Maranon and Reckelhoff, 2013). All analyzed rats were at the end of the aging phase (17–18 months old) in the time of heart excision. The endpoint selected for imaging and subsequent quantitative analysis of the microstructural remodeling corresponds to early systolic failure, which is a distinct disease stage of the hypertensive heart disease in humans as well (LeGrice et al., 2012). The animals were bought from the Charles River Laboratories (Wilmington, MA, USA). All animal procedures conformed to the guidelines set forth by the Animal Welfare Research Committee of Lawrence Berkeley National Laboratory.

### 2.2. Heart Preparation

Under deep isoflurane-inhalation anesthesia, each intact heart was rapidly removed from the chest and flushed with warmed isotonic saline. Once the heart was rinsed, it was weighed and instantaneously placed in 60cc of 10% buffered formalin (Sigma-Aldrich Corp., St. Louis, MO, USA) to induce tissue fixation at a contraction state. All hearts of this study were approximately in the same state of contraction as confirmed by measurements of the mean ventricular wall thickness (Omann et al., 2019). The whole-body weight before excision was also recorded. The period between harvest and imaging was approximately 1 week, following the advice on cardiac specimen preparation by Giannakidis et al. (2016a). Before euthanasia, and as part of a separate study (Hernandez et al., 2013), the living rats were imaged using a small animal PET/CT scanner (Inveon dedicated PET docked with CT in the multi-modality platform; Siemens Medical Solutions) and the ^18^F-FDHROL radiotracer to evaluate the left-ventricular ejection fraction (EF). EF was measured using the clinical cardiac software Myovation designed for the Xeleris workstation (GE Healthcare).

### 2.3. Imaging

Whole heart imaging was carried out at the Small Animal MRI Imaging Facility of the University of Utah using a 7T horizontal bore MRI scanner (Bruker BioSpin, Ettlingen, Germany) interfaced with the Paravision 5.1 software package. All MRI acquisitions were performed at typical room temperature, approximately 20°C. For imaging, each heart was placed in a sealed 5-mL standard syringe filled with Fomblin (Solvay Solexis, West Deptford, NJ, USA) to increase contrast and eliminate susceptibility artifacts near the boundaries of the myocardium. Hearts were secured inside the containers using gauze to prevent the specimen from floating in the container. The long axis of each heart was aligned with the *x*-axis of the scanner. A custom-made radio frequency (RF) coil (single turn solenoid, wrapped around the syringe) was used for signal transmission and reception.

To acquire the DT-MRI data, a standard 3D spin echo readout sequence was used with the following parameters: echo time (TE) = 19.224 ms, pulse repetition time (TR) = 500 ms. Diffusion encoding was carried out using a pair of trapezoidal gradient pulses with parameters: gradient duration (δ) = 4 ms, inter-gradient separation (Δ) = 10 ms, rise time = 0.25 ms, maximum strength of the gradient pulse (*G*) = 30 G/cm, resulting in a nominal *b* value of 1,000 s/mm^2^, accounting for all imaging gradients and cross-terms between imaging and diffusion gradients. The diffusion-induced signal decay was measured along 12 optimized (Papadakis et al., 1999) directions. All diffusion measurements were preceded by the acquisition of one reference (B0) image. The number of signal averages that was acquired was one and the total scan duration for each heart was approximately 17 h. The field of view (FOV) was 27 × 15.5 × 15.5 (mm), with a data matrix size = 169 × 97 × 97, resulting in an isotropic resolution of 0.160 mm.

The SNR was measured in the B0 image of each dataset as the mean signal of the myocardium in the central slice of the dataset divided by the standard deviation of the noise (measured outside the myocardium), multiplied by the factor 0.655

(1) $$\begin{array}{r} SNR_{\left(b=0\right)}=0.655\frac{Mean\left(myocardium\right)}{Standard\;Deviation\left(Background\;Noise\right)} \end{array}$$

where the factor 0.655 was used to account for the fact that the SNR was estimated on the magnitude data and the mean of the noise is not zero (Rician noise) (Henkelman, 1985).

### 2.4. Data Analysis

**Figure 1:**
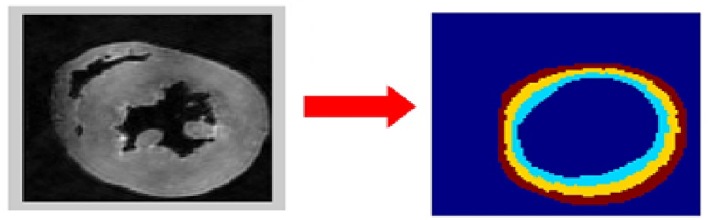
Determination of the region of interest (ROI). The left ventricular wall was segmented semi-automatically in the B0 dataset (left image). Extra care was taken to exclude papillary muscles. The three transmural sectors (endocardium in cyan, mesocardium in yellow, epicardium in brown) were defined evenly spanning the left ventricular wall (right image).

Five short-axis slices, evenly-spaced between the left ventricular base and apex, were analyzed for each heart. Endocardial and epicardial contours were semi-automatically acquired in the B0 images using cubic splines (de Boor, 1978). Extra care was taken to exclude papillary muscles. To assess whether the left ventricular cardiomyocytes respond uniformly to their hypertensive environment, each profile was divided into three transmural zones of equal thickness (i.e., epicardium, mesocardium, endocardium) (Figure 1).

The rank two symmetric diffusion tensors were estimated on a pixel-by-pixel basis over the myocardial tissue region using the diffusion signal attenuation data, the reference data, and a custom-made multi-variate nonlinear least-squares curve fitting algorithm (Hsu et al., 1998). Following this, the tensor at each voxel was diagonalised to yield the three eigenvalues *d*_1_, *d*_2_, *d*_3_ (where *d*_1_ ≥ *d*_2_ ≥ *d*_3_), and the corresponding eigenvectors ***e***_1_, ***e***_2_, ***e***_3_. The eigenvectors represent the three principal axes of diffusion, whereas each eigenvalue is equal to the rate of diffusion along the direction that the paired eigenvector points. Therefore, any voxel, at which the diffusion tensor diagonalisation resulted in at least one negative eigenvalue, was excluded on thermodynamic grounds. The longitudinal diffusivity (λ_*L*_), transversal diffusivity (λ_*T*_), mean diffusivity (MD) and fractional anisotropy (FA) were subsequently derived from the three eigenvalues using the following formulae, respectively:

(2) $$\begin{array}{r} \lambda_{L}=d_{1} \end{array}$$

(3) $$\begin{array}{r} \lambda_{T}=\frac{d_{2}+d_{3}}{2} \end{array}$$

(4) $$\begin{array}{r} MD=\frac{d_{1}+d_{2}+d_{3}}{3} \end{array}$$

(5) $$\begin{array}{r} FA=\sqrt{\frac{3\left[{\left(d_{1}-MD\right)}^{2}+{\left(d_{2}-MD\right)}^{2}+{\left(d_{3}-MD\right)}^{2}\right]}{2\left[{\left(d_{1}\right)}^{2}+{\left(d_{2}\right)}^{2}+{\left(d_{3}\right)}^{2}\right]}}. \end{array}$$

FA is a measure that describes the degree of deviation of a diffusion tensor from the isotropic tensor. In addition, since it was shown (Hsu et al., 1998) that the primary eigenvector of the diffusion tensor coincides with the local cardiomyocyte orientation, then, λ_*L*_ represents the water diffusivity parallel to the long axes of cardiomyocytes, while λ_*T*_ corresponds to water diffusivity perpendicular to the axonal myocardial cells. λ_*L*_ is considered to represent cell axonal integrity, whereas λ_*T*_ is regarded as a measure of cell integrity along its diameter (myelin integrity). To quantitatively compare the differences in the above parameters between the two groups, the mean values were computed for each of the three transmural zones across the five short-axis slices.

**Figure 2:**
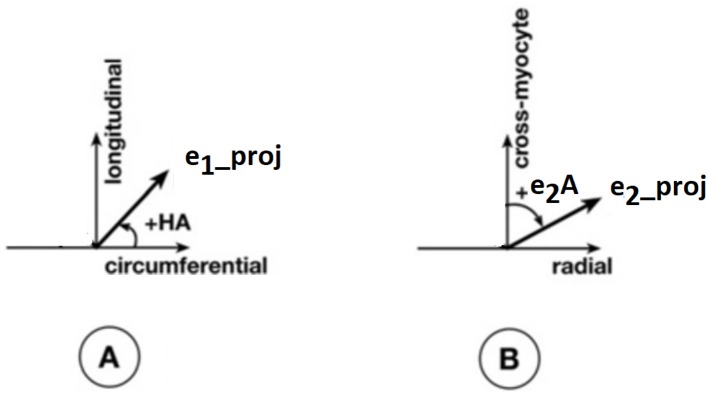
Definitions of helix angle (HA) and angle of the secondary eigenvector of diffusion relative to the local wall tangent. **(A)** HA is the angle between the circumferential direction (which is tangential to the wall with a counter-clockwise direction when viewed from base to apex) and the radial projection of the primary eigenvector *e*_1_ to the local wall tangent. HA takes values in the range −90° to 90°, being positive (right-handed helix) if rotated counter-clockwise from the circumferential as viewed from the outside, and negative (left-handed helix) if rotated clockwise. The longitudinal direction is parallel to the left-ventricular long-axis and pointing toward the base. **(B)** To calculate the angle of the secondary eigenvector of diffusion relative to the local wall tangent, we first calculated the cross-myocyte plane as the one being perpendicular to the primary eigenvector. The pertinent angle is then given by the angle between the projection of the secondary eigenvector *e*_2_ onto this plane and the cross-myocyte direction. This angle takes values in the range [−90°, 90°], being positive if rotated clockwise from the cross-myocyte direction when viewed in the more circumferential direction, and negative if rotated counter-clockwise. The radial direction is obtained by the cross-product of the circumferential and longitudinal directions, described above, pointing outwards.

Apart from the degree of anisotropy and magnitude of water diffusion, its orientation was also studied. To this end, helix angle (HA) maps were determined, where HA is defined (Scollan et al., 1998) as the angle between the cardiac short-axis plane and the projection of the primary eigenvector onto the epicardial tangent plane (Figure 2). Such maps have been frequently employed to characterize the classic helix-like orientation pattern of the aggregated cardiomyocytes within the left ventricular wall. To quantitatively compare the differences in HAs between the two groups, we computed the percentages of left-handed cardiomyocytes (i.e., cells with HAs between −90° and −30°), circumferential cardiomyocytes (i.e., cells with HAs between −30° and 30°), and right-handed cardiomyocytes (i.e., cells with HAs between 30° and 90°) for our region of interest (ROI). Finally, assuming (Nielles-Vallespin et al., 2017) that the cross-myocyte components of water diffusion are constrained by the laminar microstructures of the myocardium, we estimated the angles of the secondary eigenvector of diffusion relative to the local wall tangent plane (Figure 2), as described in Ferreira et al. (2014) for both control and diseased hearts.

All comparison tools that were employed in this study conform to previous findings (Giannakidis et al., 2016b) on the tensor manifold for cardiac DT-MRI data.

### 2.5. Statistical Analysis

To test the statistical significance of the differences in the mean parameter values between the two groups, the nonparametric Mann–Whitney test was employed. To assess dissimilarities in the HA distributions between the SHRs and WKYs, we used the chi-squared test for independence. A value of *p*<0.05 was considered statistically significant.

All computations described in section 2 were performed using in-house code written in Matlab (Mathworks, Natick, MA, USA).

## 3. Results

The average heart-to-body weight ratio was 0.6550% for the SHRs vs. 0.3450% for the WKYs (*p* = 0.0286), confirming the development of left ventricular hypertrophy in the SHRs at the time of our experiments. The mean SNR for the B0 images was approximately 90 in both groups. The average EF was 69% for the WKYs and 50% for the SHRs, confirming that the SHRs were in borderline heart failure stage.

**Figure 3:**
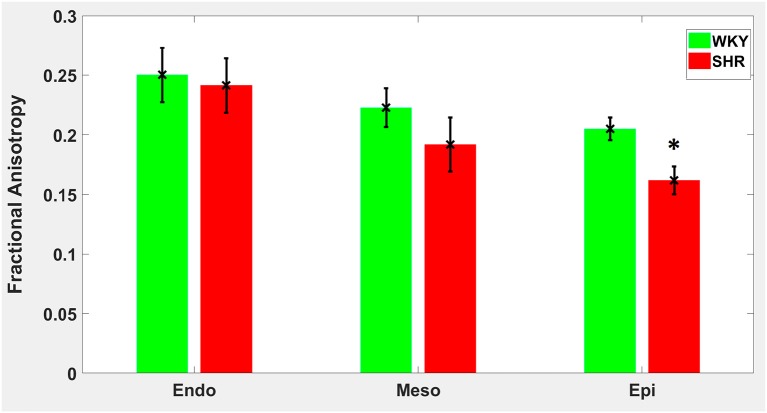
Fractional anisotropy (FA) average in each group for the three transmural zones (i.e., endocardium, mesocardium, epicardium). Every zonal average is represented by a bar graph with corresponding standard deviations as error bars. ^*^p < 0.05 compared with controls.

**Figure 4:**
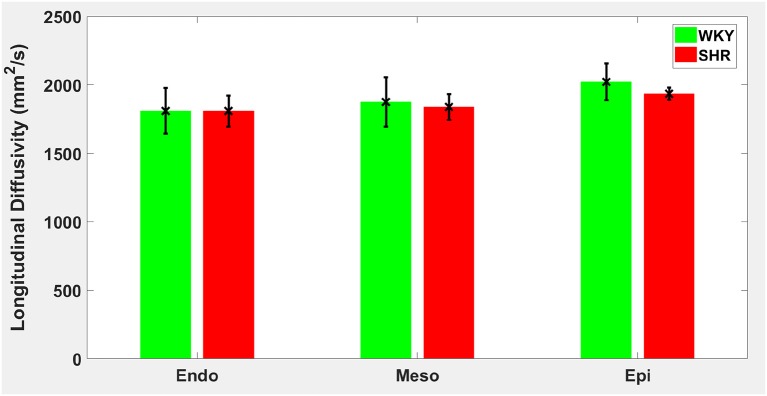
Longitudinal diffusivity (λ_*L*_) average in each group for the three transmural zones (i.e., endocardium, mesocardium, epicardium). Every zonal average is represented by a bar graph with corresponding standard deviations as error bars. The unit of λ_*L*_ is mm^2^/s.

**Figure 5:**
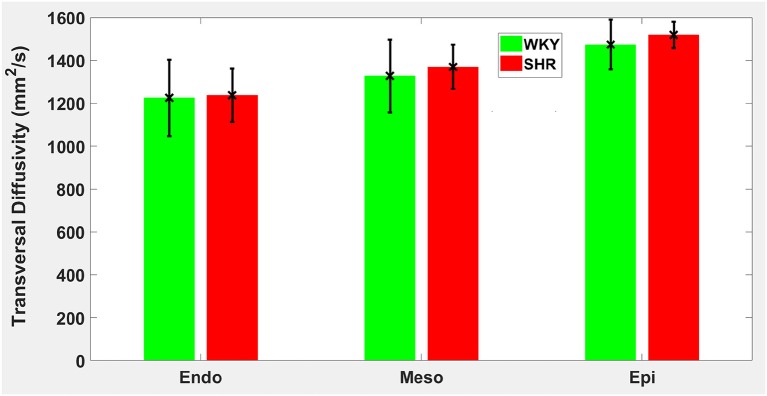
Transversal diffusivity (λ_*T*_) average in each group for the three transmural zones (i.e., endocardium, mesocardium, epicardium). Every zonal average is represented by a bar graph with corresponding standard deviations as error bars. The unit of λ_*T*_ is mm^2^/s.

**Figure 6:**
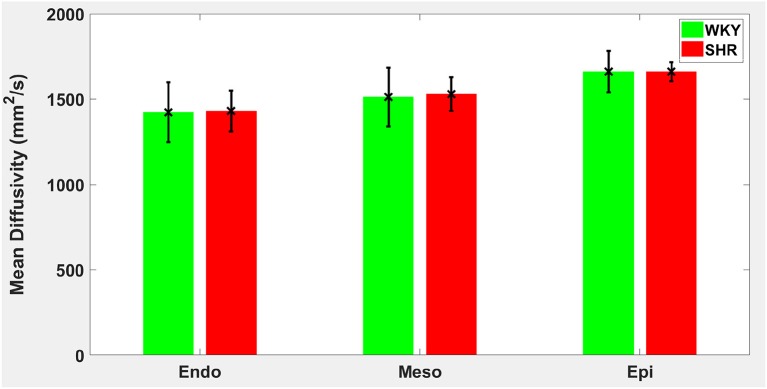
Mean diffusivity (MD) average in each group for the three transmural zones (i.e., endocardium, mesocardium, epicardium). Every zonal average is represented by a bar graph with corresponding standard deviations as error bars. The unit of MD is mm^2^/s.

Hypertension gave rise to a statistically significant decrease in epicardial FA (*p* = 0.0286), when compared to controls (Figure 3). The same remodeling was observed in the other two transmural zones, but the changes did not reach the statistical significance level (Figure 3). No statistically significant differences were found in the mean λ_*L*_, λ_*T*_, and MD values between the two groups (Figures 4–6), even though the mean λ_*T*_ and MD values were generally larger in SHRs than the controls (Figures 5, 6). By performing Pearson coefficient analysis, it turned up that there was no significant correlation (*p* = 0.3615) between the degree of cardiac hypertrophy and epicardial FA. A summary of the quantitative comparison results with respect to the degree of anisotropy and magnitude of water diffusion is presented in Table 1.

**Table 1:** A summary of the quantitative results of the comparison between WKYs and SHRs with respect to the degree of anisotropy and magnitude of water diffusion.

		**WKY**	**SHR**	***p*-value**
		**mean ± std**	**mean ± std**	
FA	endo	0.2502 ± 0.0228	0.2413 ± 0.0228	0.3429
	meso	0.2226 ± 0.0162	0.1918 ± 0.0226	0.1143
	epi	0.2050 ± 0.0095	0.1619 ± 0.116	0.0286
**λ_*L*_**	endo	1,811 ± 166	1,809 ± 114	1.0000
	meso	1,875 ± 180	1,839 ± 94	1.0000
	epi	2,022 ± 133	1,935 ± 44	0.3429
**λ_*T*_**	endo	1,225 ± 178	1,237 ± 123	1.0000
	meso	1,327 ± 170	1,370 ± 103	0.4857
	epi	1,474 ± 117	1,519 ± 62	0.4857
MD	endo	1,423 ± 174	1,431 ± 121	1.0000
	meso	1,513 ± 173	1,529 ± 98	0.6857
	epi	1,661 ± 122	1,661 ± 56	0.8857

*All parameter values are expressed as group averages for the specific transmural zone ± standard deviations. FA is fractional anisotropy, λ_*L*_ is longitudinal diffusivity, λ_*T*_ is transversal diffusivity, MD is mean diffusivity. All diffusivity values are in mm^2^/s*.

**Figure 7:**
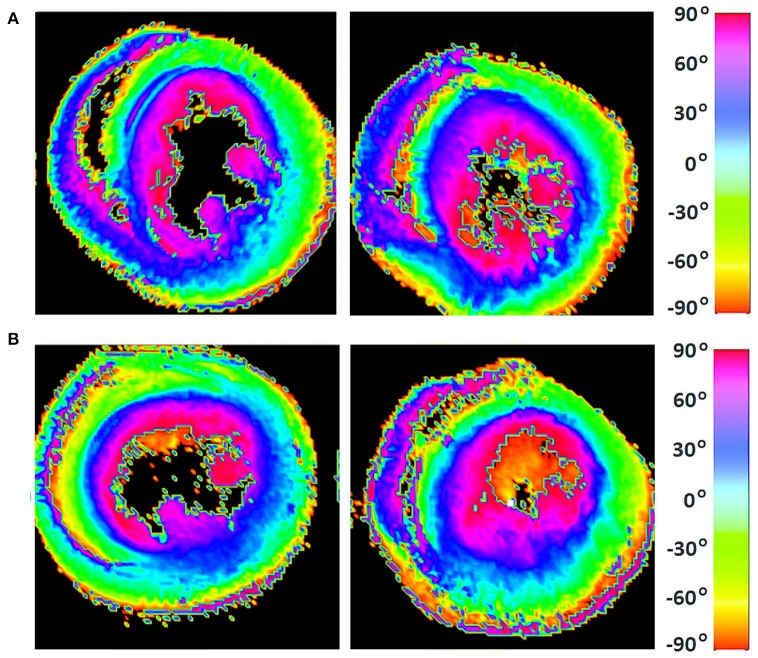
Visualization of helix angle (HA) maps from **(A)** two representative WKYs, **(B)** two representative SHRs. The displayed short-axis slices are from the equatorial region of each heart.

**Figure 8:**
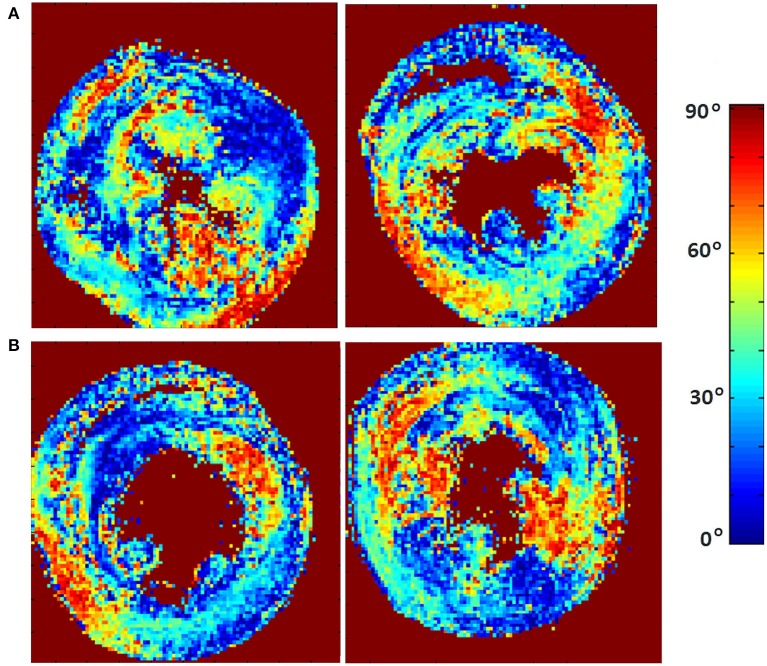
Visualization of the absolute angle maps of the second eigenvector of diffusion relative to the local wall tangent from **(A)** two representative WKYs, **(B)** two representative SHRs. The displayed short-axis slices are from the equatorial region of each heart.

The classic transition of HAs (namely, from negative values at the epicardium to zero at the mesocardium to positive values at the endocardium) was preserved after hypertension (Figure 7). By running the chi-squared test for independence on the HA distributions, we found that we cannot reject the null hypothesis that there is no association between HA distribution and status (*p* = 0.9829) (Table 2). Finally, a consistent distribution pattern of the angles of the secondary eigenvector with respect to the local wall tangent plane was not identified for either group (Figure 8).

**Table 2:** A summary of the quantitative results of the comparison between WKYs and SHRs with respect to helix angle (HA) distribution.

	**LHC**	**CC**	**RHC**
	**mean ± std**	**mean ± std**	**mean ± std**
WKYs	16.7183 ± 3.1028	56.1480 ± 3.1986	27.1195 ± 2.1782
SHRs	16.6137 ± 3.9436	55.0974 ± 7.1503	28.2771 ± 5.3936
*p*-value		0.9829	

*All parameter values are proportions (%) and are expressed as group averages for the specific orientation band ± standard deviations. LHC is left-handed cardiomyocytes, CC is circumferential cardiomyocytes, and RHC is right-handed cardiomyocytes. The observed chi-squared test statistic is greater than the critical value*.

## 4. Discussion

Unrelenting high blood pressure brings the left ventricle to a pathological pressure overload state which, when left uncontrolled, mediates cardiac remodeling and dysfunction (Weber et al., 1987; Crozatier and Hittinger, 1988; Yazaki et al., 1989). In this paper, our aim was to use the SHR animal model and DT-MRI to assess the transmural layer-specific remodeling of cardiac microstructure associated with hypertension.

This is the first exhaustive and unbiased group study of the cardiac microstructural reorganization in the SHR using DT-MRI. A previous cardiac DT-MRI study (Giannakidis et al., 2013) compared only one SHR and one WKY. Another related study (Tran et al., 2016) examined only the tissue orientation in SHRs, however, its results are biased in the sense that the same healthy (WKY) hearts were used both for creating the atlas and for the atlas-based comparison with the newly added SHRs.

A main finding of our study was that hypertension gave rise to a statistically significant decrease in epicardial FA, when compared to controls. The same remodeling trend was observed in the other two transmural zones, but the changes did not reach the statistical significance level. Such a transmural inhomogeneity of microstructural remodeling is in line with an early laborious tissue morphometry study (Engelmann et al., 1987) of the SHR model that reported on the presence of distinct, small foci of necrotic cells that were more pronounced in epicardial regions compared to other transmural layers. The transmural layer-specific cellular necrosis ensues as a result of the fact that cardiomyocytes at the outer layer of the heart are less protected from damage, compared to endocardial and mesocardial cells, due to their increased distance from the oxygen diffusing chamber following hypertensive ventricular hypertrophy (Engelmann et al., 1987; LeGrice et al., 2012). Areas of necrotic tissue are typically consistent with lower FA values, as validated in cerebral infarction (Pierpaoli et al., 1993). This diminished number of cardiomyocytes might act as a mechanism involved in the loss of contractile mass and function when hypertensive heart disease patients transition from adaptation to heart failure (Gonzalez et al., 2006; Díez and Frohlich, 2010). In general, decreases in FA can be multifactorial with swollen cardiomyocytes, cellular disarray, increases in collagen etc. having important roles and, indeed, similar FA decreases have been manifested in other cardiac pathologies such as infarction (Wu et al., 2006), dilated cardiomyopathy (Li et al., 2009), and hypertrophic cardiomyopathy (Tseng et al., 2006). A further observation of this study was that no substantial differences in diffusivity values were detected between SHRs and their normotensive equivalents.

With regards to tissue orientation, we found that the classic transmural transition of HAs was preserved after hypertension. A further quantitative analysis showed that there was no association between HA distribution and health status. This result is in agreement with a focal microscopy study (Pope, 2011) that reported insignificant differences in the transmural gradients of cardiomyocyte orientations between WKYs and SHRs.

Lastly, a consistent distribution pattern of the angles of the secondary eigenvector with respect to the local wall tangent plane was not identified for either group. This might be due to the overly complex structure of the myocardial laminar sheetlets that necessitate longer diffusion time. Another possible interpretation is that the simplistic unimodal Gaussian diffusion assumption made by DT-MRI does not satisfy the sheetlet imaging demands, and more advanced diffusion protocols, such as Q-ball imaging (Tuch, 2004), should be used when trying to elucidate this highly impenetrable structure. Further investigation of this finding is required.

The progression of hypertensive heart disease toward heart failure is a complex and multifaceted process. As well as changes in cardiac microstructure, many other pathogenetic mechanisms have been held responsible for the adverse effect on cardiac function (Brilla, 1994). These include metabolic abnormalities, interstitial remodeling, and changes in neurohormonal systems. In a longitudinal PET study (Hernandez et al., 2013) carried out using the same animal cohort as the present study, the SHRs exhibited statistically significant alterations in myocardial substrate metabolism characterized by increased glucose and fatty acid utilizations as early as in 8 months of age, followed by an increased reliance on glucose metabolism with the advancement of hypertrophy and heart failure. These changes were correlated with increases in blood flow and alterations in overall cardiac function. The findings of the present study are in line with these results.

Our results are of great clinical relevance. The manifested transmural nonuniformity of the response of rodent myocardial microstructure to hypertension suggests that there is a necessity to assess the cardiomyocyte arrangement (not only at numerous time points but also) at various depths across the left ventricular wall in order to fully understand and characterize the behavior of the whole organ in hypertension.

The fact that the imaging experiments of this investigation were performed when the SHRs were at the early systolic failure stage of the hypertensive heart disease hints that our findings could also be used to help identify those patients for whom it is required to adopt therapeutic strategies that prevent the progresion toward heart failure. Examples of such treatment might be the blunting of intracellular apoptotic pathways and the stimulation of cellular survival mechanisms that slow down the cell death processes (Gonzalez et al., 2006).

Computer-based electromechanical modeling of the heart at multiple scales is being extensively used in the literature (Markhasin et al., 2003; Vadakkumpadan et al., 2010; Sermesant et al., 2012) in order to better understand the function of this complex organ. Given that a major goal of these modeling studies is to accurately simulate pathology, our findings are expected to be important for investigating the mechanisms for the cardiac function degradation in hypertensive heart disease. These mechanisms remain poorly understood to date (Wang et al., 2016). Considering that one needs to account for myocardial microstructure changes to explain differences in chamber compliance, our high-resolution results could be used for the development of more realistic subject-specific constitutive modeling frameworks toward determining the role that microstructural remodeling plays in the passive mechanical function of the heart during the progression of this disease (Wang et al., 2015, 2016). In addition, our results might be important for modeling studies of cardiac electrophysiology (Franzone et al., 1998; Muzikant and Henriquez, 1998) such as, for example, when one tries to determine the organ's predisposition to arrhythmia while still in the compensated state of hypertension (Evans et al., 1995).

This study has few limitations. At first, there is a lack of validation of our findings through histology. Nevertheless, the revealed differences were consistent with findings from early laborious cellular morphometry studies on the same animal model. Next, the small number of animals in both groups is also a weakness. However, the distinctness of the hypertension effects on epicardial FA was highlighted by the observed statistically significant differences between the two groups. The use of formalin fixation may also form a limiting factor in imaging studies. Our chosen duration of tissue exposure to formalin before imaging nonetheless does not appear to amend the DT-MRI parameters when compared to *in vivo* imaging (Giannakidis et al., 2016a). The fact that an animal model was used may also be viewed as a caveat, and a cautious approach is mandatory when findings in animal models are being extrapolated to human hypertension. However, we believe that the differences between rat and human physiology do not adversely affect our goal, since previous results showed (Lerman et al., 2005) that the SHR model mimics many of the pathophysiology seen in hypertensive heart disease in humans. Another shortcoming of this paper was that only aged (17–18 months old) rats were analyzed. A future DT-MRI study should investigate more phases of the SHR remodeling to shed more light on the time course of hypertensive heart disease. An inherent error of our imaging technique is that a voxel volume of 0.160 mm^3^ was sampled. Bearing in mind the average cardiomyocyte size of 20 × 20 × 80 μm^3^ (Okabe et al., 1999), this means that the primary eigenvector in each voxel of our study represents the mean direction of approximately 128 cardiomyocytes. Noise is another source of error in MRI experiments. However, due to the very high SNR achieved in our experiments, it is unlikely that noise has caused any bias. ROI measurements can also induce errors related to reproducibility. Finally, another limitation of this study is that we have not included any measurements of the haemodynamic state of the animals before they were killed. In fact, we tried early in our study to obtain blood pressure measurements of the rats using an external non-invasive method (Ohta et al., 2017). However, we found that this was very difficult, and a lot of effort and preparation time was required in order to maintain the correct environment which was necessary to ensure the accuracy of the small animal data. We actually obtained high blood pressure readings (> 150 mm Hg) for the SHR model, but it was difficult to know if the readings reflected the true blood pressure since the animals exhibited significant stress during the measurement (Jamieson et al., 1997). Likewise, the invasive method to obtain blood pressure measurements in small animals has many disadvantages despite being more accurate (Parasuraman and Raveendran, 2012). In this case, anesthesia is required to perform catheterization which may also lead to haemodynamic changes. In addition, it is a very laborious process, as it necessitates tracheostomy and cannulation of carotid artery. Therefore, we decided on not including any blood pressure measurements in this study. There is nevertheless abundant evidence in the literature (Kawamura et al., 1976) that the 17–18 month old SHRs have long-standing high blood pressure (their blood pressure is significantly higher than in the controls since 7 weeks old). Accordingly, we cannot tell whether more hypertension related to larger decreases in epicardial FA. However, we found that there was no significant correlation between the degree of cardiac hypertrophy and epicardial FA.

In summary, the transmural microstructural nonuniformity of the rodent myocardial response to hypertension gave rise to measurable differences in the DT-MRI-derived FA. The epicardium is more vulnerable to high blood pressure leading to more pronounced microstructural alterations in this region during remodeling. Our observations corroborate previous labor-intensive cellular morphometry studies. Spatial factors are important for fully understanding the heart's behavior in hypertension. Our findings could be useful for the improved management of patients with systemic arterial hypertension, thus prevent further damage. The results of this study are timely and their incorporation in models of cardiac electrophysiology and biomechanics may lead to more robust frameworks that simulate this pathology.

## Data Availability Statement

The datasets generated for this study can be found in figshare repository (https://figshare.com/articles/Ex_vivo_cardiac_DT-MRI_rat_study_-_LBNL/11663301).

## Ethics Statement

The animal study was reviewed and approved by Animal Welfare Research Committee of Lawrence Berkeley National Laboratory.

## Author Contributions

GG conceived and designed the study, and wrote the paper. AG analyzed the data, wrote the software used in analyses, and wrote the paper. All authors agree to be accountable for the content of the work.

### Conflict of Interest

The authors declare that the research was conducted in the absence of any commercial or financial relationships that could be construed as a potential conflict of interest.

## Footnotes

^1^https://www.nhs.uk/conditions/high-blood-pressure-hypertension/

^2^https://www.who.int/gho/ncd/risk_factors/blood_pressure_prevalence_text/en/

^3^https://www.who.int/features/qa/82/en/
